# Targeting Long Non-Coding RNAs in Hepatocellular Carcinoma: Progress and Prospects

**DOI:** 10.3389/fonc.2021.670838

**Published:** 2021-06-25

**Authors:** Xinrong Lin, Xiaosong Xiang, Bing Feng, Hao Zhou, Ting Wang, Xiaoyuan Chu, Rui Wang

**Affiliations:** ^1^ Department of Medical Oncology, Affiliated Jinling Hospital, Medical School of Nanjing University, Nanjing, China; ^2^ Affiliated Jingling Hospital Research Institution of General Surgery, School of Medicine, Nanjing University, Nanjing, China

**Keywords:** long non-coding RNA, hepatocellular carcinoma, signaling pathway, therapy resistance, clinical transformation

## Abstract

Hepatocellular carcinoma is the fifth-ranked cancer worldwide with a relatively low five-year survival rate. Long non-coding RNAs are a group of RNAs with remarkable aberrant expression which could act on multiple bioprocesses and ultimately impact upon tumor proliferation, invasion, migration, metastasis, apoptosis, and therapy resistance in cancer cells including hepatocellular carcinoma cells. In recent years, long non-coding RNAs have been reported to be indispensable targets in clinical target therapy to stop the growth of cancer and prolong the lifespan of patients with hepatocellular carcinoma. In this review, we enumerate the signaling pathways and life activities affected by long non-coding RNAs in hepatocellular carcinoma cells to illustrate the role of long non-coding RNAs in the development and therapy resistance of hepatocellular carcinoma.

## Introduction

Liver cancers comprise diverse, histologically distinct primary hepatic neoplasms, which include hepatocellular carcinoma (HCC), intrahepatic bile duct carcinoma (holangio carcinoma), hepatoblastoma, bile duct cystadenocarcinoma, hemangiosarcoma, and epitheliod hemangioendothelioma (1). Among them, HCC is one of the most prevalent liver cancers worldwide (2, 3). The main risk factors for HCC vary from region to region. Chronic hepatitis B virus (HBV) infection and AFB_1_ exposure are major risk factors in most high-incidence regions like Asia and Africa. In contrast, HCV infection, excessive drinking, and diabetes/obesity/metabolic syndrome play a more important role in low-incidence areas, with the primary risk factor being HCV infection. In addition, some hereditary metabolic disorders such as hemochromatosis, *α*_1_ anti-trypsin deficiency, tyrosinemia, and several porphyrias also increase the risk (4–8). Till now, lots of treatments for HCC such as resection, ablation, transplantation, chemoembolization, and targeted drugs like sorafenib and lenvatinib are put into clinical practice (9–11). Despite all those treatments, the current five-year survival rate provided by the National Cancer Institute is still not satisfactory, so we still devote a lot of effort to create more effective methods (12). LncRNAs which are aberrant expressions are proved to be associated with tumorigenesis and poor prognosis of HCC. Given that, lncRNAs are promising potential biomarkers or targets of HCC.

LncRNAs belong to the ncRNA class, which consists of small interfering RNAs (siRNAs), microRNAs (miRNAs), PIWI-interacting RNAs (piRNAs), promoter-associated RNAs (PARNAs), small nucleolar RNAs (snoRNAs), and other recently described classes such as X-inactivation RNAs (xiRNAs), sno-derived RNAs (sdRNAs), microRNA-offset RNAs (moRNAs), tRNA-derived RNAs and MSY2-associated RNAs (MSY-RNAs) (13). LncRNAs are approximately 200 bp to 100 kb in length (14). Although most lncRNAs are not directly involved in gene coding and protein synthesis, they are indispensable in genomic imprinting, chromatin modification, post-transcriptional regulation, cleavage and modification (15–17). We screened lncRNAs in human HCC cells by using EVLncRNAs (a database) (**Table 1**) and selected some well-studied lncRNAs to describe in detail. Some well-known regulatory pathways of lncRNAs in HCC have also been demonstrated (**Table 2**).

**Table 1:** Human lncRNAs related to HCC (EVLncRNAs).

	LncRNAs
Up-regulated	*BANCR, BC014579, CCAT1, CDKN2B-AS1, CYTOR, DANCR, DBH-AS1, EGFR-AS1, FTX, GAS5, H19, HEIH, HELLS, HOTAIR, HOTTIP, HOXA13, HULC, ICR, LINC00974, LINC01225, LINC01419, LINC01419, lnc-AF085935, lncRNA-ATB, lnc-uc003wbd, MALAT1, MEG3, NEAT1, PANDAR, PCNA-AS1, PVT1, RP11-160H22.5, RP11-401P9.4, RP11-501G6.1, RP11-672F9.1, RP5-1014O16.1, SIRT1-AS, SIRT1-AS 622C mutation, SNHG19, SNHG3, SOX2-OT, TUG1, uc004bdv.3, UCA1, UFC1, ULK4P2, WRAP53, WSPAR, XLOC_014172, ZEB1-AS1, ZFAS1*
Down-regulated	*AF070632, AOC4P, AX800134, C14orf132, CR613944, CTB-167B5.2, GAS5, H19, LINC00173, LINC01018, lincRNA-CALCA, lincRNA-TSPAN8, MEG3, NPTN-IT1, PRAL, PTENP1, TP53COR1, uc001ncr, WT1-AS*

**Table 2:** Dysregulated lncRNAs in HCC, their biological functions, and related molecules/pathways.

LncRNA	Full Name	Expression in HCC	Biological function	Related Molecule/Pathway	References
*ATB*	Activated by TGF-β	Up-regulated	↑EMT, invasion, metastasis, colonization	TGF-β, ZEB1, ZEB2, *miR-200*, IL-11, STAT3 pathway	(18–20)
*MALAT1*	LncRNA metastasis-associated	Up-regulated	↑EMT, proliferation, migration, metastasis, invasion	Wnt pathway, SRSF1, mTOR pathway, *miR-143-3p*, ZEB1, AJAP1, *miR-146b-5p, miR-204*, SIRT1	(21–27)
	lung adenocarcinoma transcript 1		↓Apoptosis		
*HOTAIR*	Hox transcript antisense intergenic RNA	Up-regulated	↑EMT, proliferation, invasion, metastasis, autophagy	*miR-23b-3p*, ZEB1, ATG3, ATG7, OGFr, CCND1, RBM38, *miRNA-218*, P14, and P16 signaling, GLUT1, mTOR pathway	(28–39)
*HULC*	Highly up-regulated in liver cancer	Up-regulated	↑EMT, proliferation, invasion, metastasis, autophagy	*PTEN*, miR15a, AKT–PI3K–mTOR pathway, *miR-9*-mediated RXRA signaling pathway	(25, 40–44)
*UCA1*	Urothelial cancer-associated 1	Up-regulated	↑EMT, proliferation, invasion, metastasis	Hippo signal pathway	(45–47)
*TUC338*	Transcribed ncRNA encoding uc.338	Up-regulated	↑Proliferation	PAI-1, Pax6, p53, PAI-RBP1 pathway	(29, 48)
*CCAT1*	The long non-coding RNA Colon Cancer Associated Transcript 1	Up-regulated	↑Proliferation, migration, apoptosis	*Let-7*, c-Myc, HMGA2	(49, 50)
*DANCR*	Differentiation antagonizing non-protein coding RNA	Up-regulated	↑Proliferation, metastasis	*β*-catenin,TCF/LEF	(51–53)
*HOTTIP*	HOXA transcript at the distal tip	Up-regulated	↑Proliferation	*miR-192, miR-204*, HOXA	(54–57)
*TUG1*	Taurine up-regulated gene 1	Up-regulated	↑Proliferation	*miR-142-3p*, ZEB1, *miR-144*, JAK2/STAT3 pathway	(54, 58–60)
			↓Apoptosis		
*LINC00152*	Long intergenic non-protein coding RNA 00152	Up-regulated	↑EMT, proliferation, invasion, metastasis	EpCAM, mTOR pathway, E-cadherin, EZH2	(42, 49, 61)
*MEG3*	Maternally expressed gene 3	Down-regulated	↑Apoptosis	*MEG3*, p53, MDM2	(62–64)
			↓Proliferation		
*PTENP1*	Phosphatase and tensin homolog pseudogene 1	Down-regulated	↑Autophagy, apoptosis	*PTEN*, PI3K/AKT pathway	(65–68)
			↓Proliferation, migration, invasion		
*ASLNC02525*		Up-regulated	↑Proliferation, invasion	*Hsa-miRNA-489-3p*, twist1 (twist related protein 1)	(69)
*SNHG1*	Small Nucleolar RNA Host Gene 1	Up-regulated	↑Proliferation, invasion, migration	*miR-195*	(70)
*HANR*	HCC associated long non-coding RNA	Up-regulated	↑Proliferation	GSKIP, GSK3β	(71)
			↓Apoptosis		
*Linc-USP16 (Linc00161)*	Long intergenic non-protein coding RNA 161	Up-regulated	↓Proliferation, migration	AKT pathway, *miR-21, miR-590-5p*, *PTEN*	(72)
*NEAT1*	Nuclear-enriched abundant transcript 1	Up-regulated	↑Proliferation	*miR-129-5p*-VCP-IκB system	(73)
*PCAT-1*	Prostate cancer-associated transcript 1	Up-regulated	↑Proliferation	*miR-215*, CRK-like proto-oncogene, adaptor protein (CRKL)	(74)
*Lnc-EGFR*	Lnc-epidermal growth factor receptor	Up-regulated	↑Proliferation	EGFR/Foxp3, AP-1/NF-AT1 axis	(75)
*MVIH*	LncRNA associated with microvascular invasion in HCC	Up-regulated	↑Proliferation, migration	ARID1A, SWI/SNF chromatin remodeling complex	(75)

## FUNCTIONS of lncRNAs in HCC

### LncRNAs and miRNAs

#### LncRNAs Acting as miRNA Sponges or miRNA Inhibitors

*MALAT1* (*LncRNA metastasis-associated lung adenocarcinoma transcript 1*) binds to and inhibits *miR-143-3p* expression to decrease ZEB1 (zinc finger E-box binding home box 1) (21). Also, *MALAT1* acts as molecular sponge of *miR-146b-5p* and *miR-204* to facilitate HCC cells (22, 23). The *UCA1* gene belongs to the HERV-H family. It contains the gag region, the protease–polymerase region, but no envelope region. In three regions, the *UCA1* full-length cDNA consists of an unusual number of stop codons that transcribe non-coding RNA (45, 46). *UCA1* facilitates FGFR1–ERK pathway by inhibiting expression of *miR-216b* (76). *CCAT1* (*The long non-coding RNA Colon Cancer Associated Transcript 1*) contains two predicted *let-7* targeting sites. It is reported that *let-7* decreases tumor proliferation and induces apoptosis. *Let-7* binds to *CCAT1* but does not induce degradation of *CCAT1*. In other words, *CCAT1* is physically related to *let-7* and serves as a miRNA sponge for *let-7*. At the same time, *CCAT1* regulates HMGA2 and c-Myc by competitively binding to *let-7* (49). *HOTAIR* (*Hox transcript antisense intergenic RNA*), which is overexpressed in HCC tissues, enhances EMT by inhibiting *miR-23b-3p*, leading to malignant tumors of HCC and increased tumor metastasis (28–30). *TUG1* is proved to act as a molecular sponge of *miR-144*. It interacts with *miR-144* to promote proliferation and migration of HCC cells by activating the JAK2/STAT3 pathway. After knocking out *TUG1* in tumor cells, the JAK2/STAT3 pathway is inactivated, and *miR-144* is up-regulated to inhibit HCC tumor growth *in vivo*. To sum up, the interaction of *TUG1* and *miR-144* promotes proliferation, migration, and tumorigenesis by activating the JAK2/STAT3 pathway in HCC (54). *PTENP1* regulates the *PTEN*/Akt pathway through interaction with miR-193a-3p (65). *HULC* promotes HCC *via* depleting *miR-9*-mediated RXRA signaling pathway (40). Overexpressed *PTENP1* induces *miR-17*, *miR-19b*, and *miR-20a*, targeting *PTEN*, PHLPP (negative AKT regulatory factor) and autophagy genes such as ULK1, ATG7, and p62 (66).

#### LncRNAs Acting as Competing Endogenous RNAs of miRNAs

LncRNA-*ATB* (activated by TGF-*β*) which is up-regulated in HCC is activated by TGF-*β* and up-regulates ZEB1 and ZEB2 by competitive binding to the *miR-200* family (77–79). ZEB1 gene encodes a zinc finger transcription factor that plays an important role in normal embryonic development which induce EMT (24). EMT converts cancerous epithelial cells into mesenchymal-like cells, confers migration and invasion properties, enables primary tumor cells to move, settles distant organs, and forms secondary tumors (18, 19, 80). As a result, the silence of ZEB1 hinders the metastasis and invasion of HCC through EMT inhibition. *TUG1* promotes HCC development by competing with *miR-132* to combine sonic hedgehog (Shh) as well as Kruppel-like factor 2 (KLF2) to combine polycomb repressive complex 2 (PRC2) (58, 59). Overexpression of *MEG3* competitively inhibits *miR-664*, thereby releasing the inhibitory effect of *miR-664* on ADH4 and promoting the expression of ADH4 (81).

#### MiRNAs Acting as lncRNA Inhibitors

*HOTTIP* (*HOXA transcript at the distal tip*) is mainly present in the nucleus, binds to AGO2 in the nucleus, and is regulated by some miRNAs. The level of *HOTTIP* is significantly decreased in cells when *miR-192*, *miR-204*, and *miR-125b* are overexpressed (82). *MiR-192* and *miR-204* inhibit the activity of *HOTTIP* through their target molecules such as DHFR, ZEB2, BCL2, and so on, thereby achieving the purpose of inhibiting the activity of HCC cells (55).

#### LncRNAs Acting on Proteins

*HOTAIR* up-regulates ATG3 and ATG7 to activate autophagy and promote HCC cell proliferation (31). In addition, OGFr (Opioid Growth Factor Receptor) which prominently impedes tumor growth is depleted in HCC because of *HOTAIR* (32). Besides, CCND1, RBM38, P14, P16, GLUT1, and mTOR signaling also participate in *HOTAIR* signaling to promote HCC progress (33–36). Meanwhile, *HULC* activates protective autophagy through Sirt1 (silent information regulator 1 protein)–USP22 (ubiquitin-specific peptidase 22) pathway and increases HCC proliferation through COX2–USP22 pathway. *HULC* reduces Sirt1 and COX2 degradation by elevating the expression of USP22 (83). *TUC338* (*Transcribed ncRNA encoding uc.338*) post-transcriptionally regulates plasminogen activator inhibitor-1 RNA binding protein (PAI-RBP1), occupying a genomic region rich in unique or known motifs homologous to the tumor suppressor Pax6 and p53 (48). C-Myc directly binds to the promoter of *CCAT1* and promotes HCC development (50). Activation of TCF/LEF by *β*-catenin is one of the most common molecular changes in HCC cells as well as a general regulator of stem cell self-renewal, tumorigenicity and tumor progression. *DANCR* (*Differentiation antagonizing non-protein coding RNA*) regulates the stability of above molecular changes to affect tumor proliferation (51, 84). *Linc00152* (*Long intergenic non-protein coding RNA 00152*) prevents E-cadherin expression by interacting with EZH2 and promotes EMT in HCC cells (61). *MEG3* (*Maternally expressed gene 3*) promotes p53 expression and inhibits MDM2 expression, and increasing p53 also inhibits the expression of MDM2. So that the ubiquitination of P53 by MDM2 is prevented, promoting apoptosis and inhibiting tumor proliferation (62, 63, 85, 86).

### Interactions Within lncRNAs

It is reported that *MALAT1*/*HULC* is positively correlated with the expression of TRF2 in human hepatocellular carcinoma tissues. *MALAT1* and TRF2 are highly expressed in HCC tissues and are positively correlated. The increased TRF2 binds to *HULC* and *MALAT1* to form a complex, which is loaded into the telomere region of the chromosome. Therefore, the telomeres are greatly extended, leading to the rapid growth of HCC stem cells (25). Overexpression of *HULC* (highly up-regulated in liver cancer) prevents *PTEN* and miR15a, which leads to high expression of LC3I and LC3II (autophagy marker) and more autophagy in hepatoma cells. *HULC* inhibits *PTEN* by autophagy and P62-mediated ubiquitin–protein system and finally activates the AKT–PI3K–mTOR pathway to promote cell growth, colony-forming ability, and cell growth *in vivo* (41).

## Roles of lncRNAs in HCC

### LncRNAs as Tumor-Suppressive Genes in HCC

*MEG3* expression is down-regulated in both HCC cell lines and tissues. Re-expression of *MEG3* in HCC cells significantly reduces anchorage-dependent and independent cell growth and induces apoptosis (64, 87). Also, adenosine can resist HCC through up-regulating the expression of *MEG3* (88).

*Phosphatase and tensin homolog pseudogene 1* (*PTENP1*) is a pseudogene of the tumor suppressor gene phosphatase and tensin homolog deleted on chromosome ten (*PTEN*) (67, 68). It is shown that *PTENP1* and *PTEN* are down-regulated in HCC cells. Over-expression of *PTENP1* and *PTEN* in HCC cells can inhibit the oncogenic PI3K/AKT pathway, inhibit cell proliferation, migration, invasion, and induces autophagy, apoptosis, and inhibition of angiogenesis.

### LncRNAs as Oncogenes in HCC

lncRNA-*ATB* promotes HCC colonization by inducing autocrine induction of IL-11 and activating STAT3 signaling (20). Plasma *Linc00152* can be used as a potential non-invasive biomarker to predict the diagnosis of HCC (42). *MALAT1* is up-regulated in HCC and plays an oncogenic role through activating the Wnt pathway and inducting oncogenic splicing factor SRSF1 to activate the mTOR pathway and resist apoptosis (26). *HOTAIR* with a length of 2,158 bp is remarkably associated with poor prognosis of HCC. It affects the histone H3 tri-methylated at lysine 27 (H3K27me3) by recruiting poly bulking inhibitor complex 2 (PRC2 complex) at the 5′ end, so that LSD1 (lysine-specific demethylase 1)/CoREST (RE1-silencing transcription factor co-repressor)/REST (RE1-silent transcription factor) complex promotes histone H3 Lysine 4 demethylation, eventually leading to gene silencing (37–39). *UCA1* (urothelial cancer associated 1) is overexpressed in HCC, making it a potential biomarker to detect progression and prognosis in patients with HCC (89). Decreasing the expression level of *UCA1* inhibits the proliferation, migration, and invasion of HCC cells and induces apoptosis. Moreover, bioinformatics analysis indicates that *UCA1* may significantly disrupt the hippo signal pathway (47). *TUC338* is a super-conservative lncRNA that contributes to the growth of transformed cells in HCC (HCC). *TUC338* functions in a manner similar to transcription factors to regulate cell proliferation and transform cell growth in HCC. Overexpression of *CCAT1* in hepatoma cells promotes proliferation, invasion, and metastasis of tumor cells (50). As a newly discovered cancer-associated lncRNA, *HOTTIP* is located at the 5′ end of the *HOXA* gene. Overexpression of *HOTTIP* could promote cell proliferation, migration, and invasion of HCC cells (54, 56, 90–92). *Linc00152* is up-regulated in the human HCC cell line. Overexpressed *Linc00152* in HCC cells increased cell proliferation and invasion. Knocking out *Linc00152* inhibits the mTOR signaling pathway. The underlying mechanism is that expression of *Linc00152* increases EpCAM levels, leading to activation of the mTOR signaling pathway and causing proliferation of HCC (49). *DANCR* positively regulates proliferation in cells by regulating *miR-634, miR-496 miR-33a-5p*, CDKN1A (cell cycle inhibitor p21), *etc*, indicating that it may be a carcinogenic lncRNA, and plays potential roles as an adenocarcinoma (ADC) biomarker and therapeutic target (52, 53). The combination of sorafenib-induced enhanced tumor growth inhibition and overexpression of RASAL1 in tumor xenografts suggests that the *TUC338*/RASAL1 axis may be a potential therapeutic target for current HCC treatment (93).

### LncRNAs and Therapeutic Sensitivity in HCC

**Figure 1:**
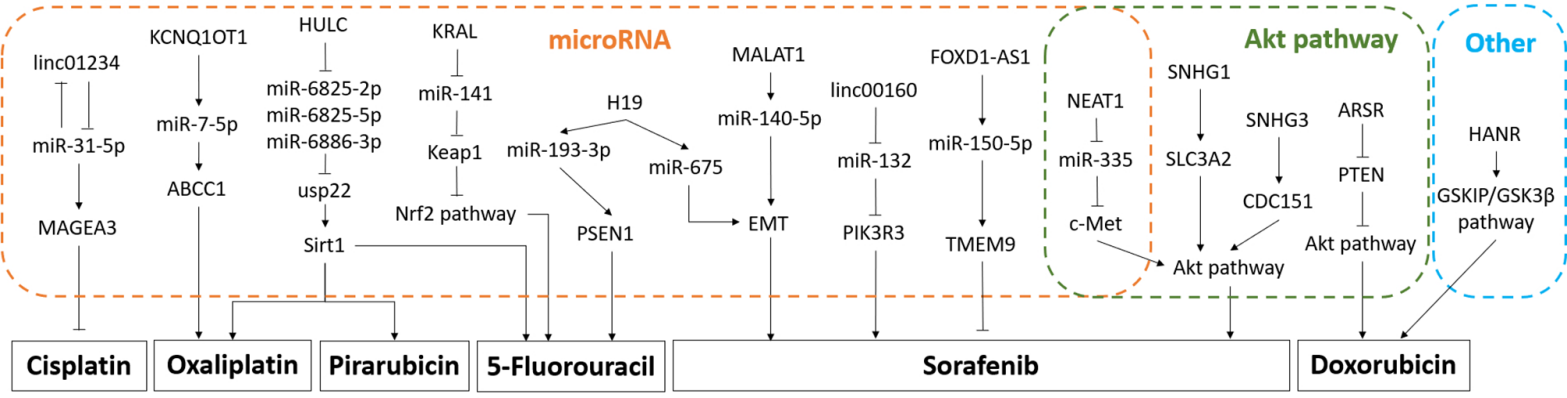
Diagram of the mechanism of lncRNA mediating chemotherapy resistance. Part of the mechanism is related to microRNAs, and part is related to Akt pathway. The promotion arrow in the panel refers to the promotion of drug resistance to the corresponding chemotherapeutic drug, and the inhibition arrow refers to the increase of the sensitivity to the chemotherapy drug.

LncRNAs not only influence HCC proliferation, invasion, and migration through specific cell signaling pathways and molecules, but also induce HCC therapy resistance, which covers chemotherapy resistance and radiotherapy resistance. It has been found that lncRNA *ROR* reduces the sensitivity of HCC to radiotherapy (94). For chemotherapy, the most commonly used chemotherapy drugs for HCC include sorafenib, oxaliplatin, 5-fluorouracil, cisplatin, *etc* (95). **Figure 1** is a schematic diagram of the relationship between lncRNAs and chemotherapeutic resistance.

#### Sorafenib

*SNHG1*, *SNHG3*, and *SNHG16* in the small nucleolar RNA host gene (*SNHG*) family are related to sorafenib resistance in HCC cells (96–98). *SNHG1* serves *miR-21* as a mediator, as well as *SNHG3* to produce positive feedback on the downstream Akt signaling pathway, and ultimately induces sorafenib resistance (99). Coincidentally, *nuclear-enriched abundant transcript 1 (NEAT1)* also acts on the downstream Akt signaling pathway through the *miR-335*/c-Met axis to induce sorafenib resistance (100). Other lncRNAs related to sorafenib resistance in HCC cells include *linc00160, FOXD2-AS1, MALAT1*, *H19, ROR*, *etc* (101). *Linc00160* promotes phosphoinositide-3-kinase regulatory subunit 3 (PIK3R3) to induce sorafenib resistance by inhibiting *miR-132* (102). *FOXD2-AS1* acts as a competitive endogenous RNA of *miR-150-5p* to increase sensitivity to drugs (103). *MALAT1* and *H19* have similar mechanisms when inducing HCC resistance, which both promote EMT through intermediary molecules to induce resistance (104–106).

#### Oxaliplatin

The lncRNAs associated with oxaliplatin resistance contain *KCNQ1OT1, HULC, and NR2F1-AS1* (107). Both *KCNQ1OT1* and *HULC* act on resistance-related molecules through microRNA, which are *miR-7-5p, miR-6825-2p, miR-6825-5p*, and *miR-6886-3p* (83, 108).

#### Doxorubicin

The lncRNAs associated with oxaliplatin resistance contain HCC associated long non-coding RNA (*HANR*) and ARSR. *HANR* triggers GSKIP/GSK3*β* pathway (71). ARSR decreases the negative molecule *PTEN* of Akt pathway (109).

#### 5-Fluorouracil

The above pathways of *HULC* also induce 5-fluorouracil and pirarubicin resistance (83). While *H19* promotes presenilin-1 (PSEN1) through *miR-193-3p*, which distinguishes from the above mentioned, to achieve 5-fluorouracil resistance (110). lncRNA *KRAL* mediates 5-fluorouracil resistance in HCC by acting as ceRNA against *miR-141* (111).

#### Cisplatin

*Linc01234/miR-31-5p*/melanoma-associated antigen A3 (*MAGEA3*) axis prompts cisplatin resistance in HCC cells when *linc01234* is overexpressed (112).

## Discussion

LncRNA-related clinical trials have not been conducted on hepatocellular carcinoma, but a clinical trial from Wuhan Union Hospital on lncRNA as a potential target for lung cancer diagnosis is underway. This clinical trial is mainly based on the identification of early lung-cancer-specific exosomal lncRNA biomarkers to improve the diagnosis rate of early lung cancer (113). Also, a clinical trial which is related to *HOTAIR* and thyroid cancer is being processed (114).

**Figure 2:**
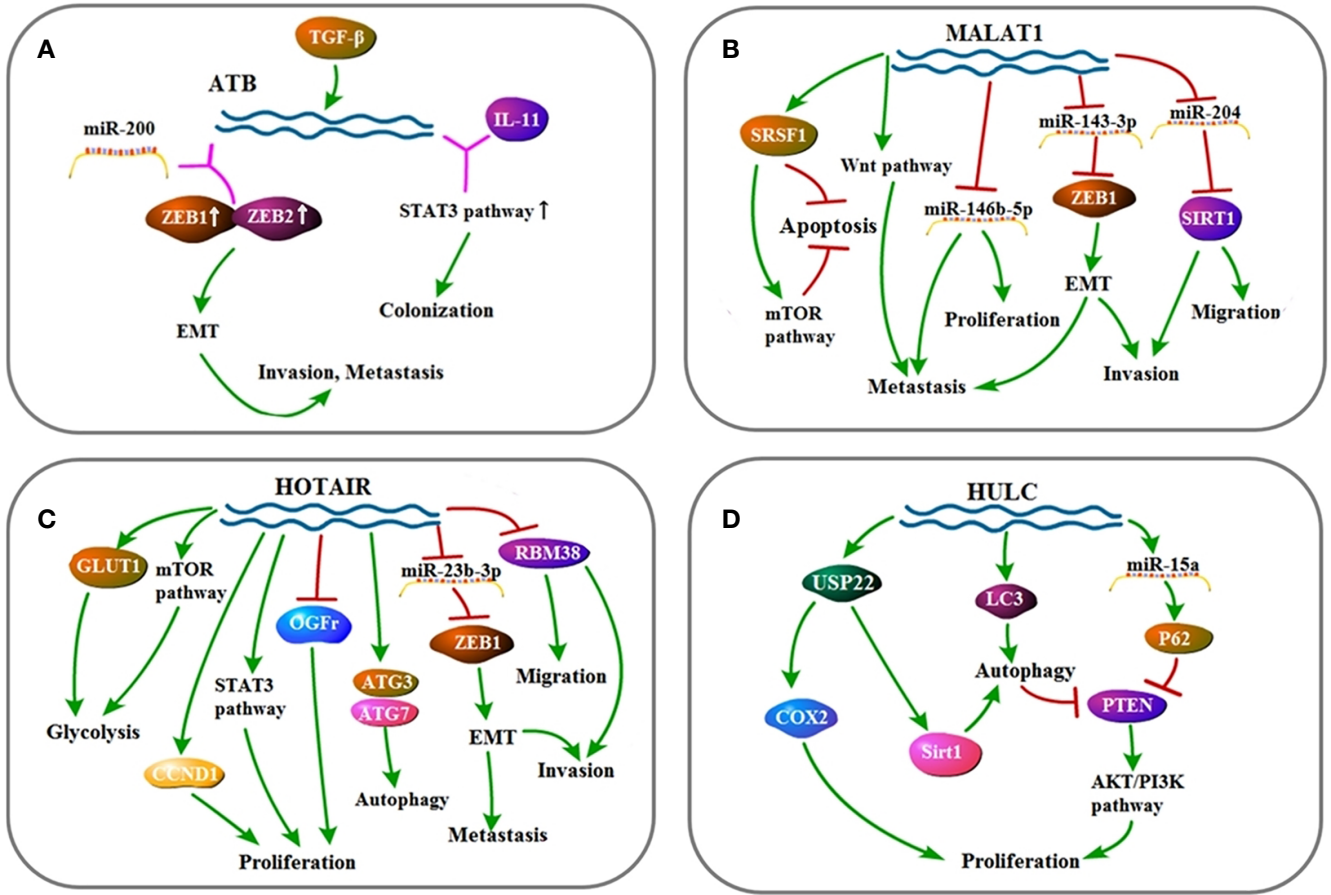
**(A)** Signaling pathway of *ATB*: *ATB* affects cell invasion, metastasis, and colonization by up-regulating EMT and STAT3 pathway. **(B)** Signaling pathway of *MALAT1*: *MALAT1* affects cell invasion, metastasis, proliferation, migration, and apoptosis mainly through up-regulating EMT, Wnt pathway, STAT3 pathway, and mTOR pathway. **(C)** Signaling pathway of *HOTAIR*: *HOTAIR* affects cell invasion, migration, autophagy, proliferation, metastasis, and glycolysis by up-regulating EMT, mTOR pathway, and STAT3 pathway. **(D)** Signaling pathway of *HULC*: *HULC* affects cell proliferation and autophagy by up-regulating AKT/PI3K pathway.

**Figure 3:**
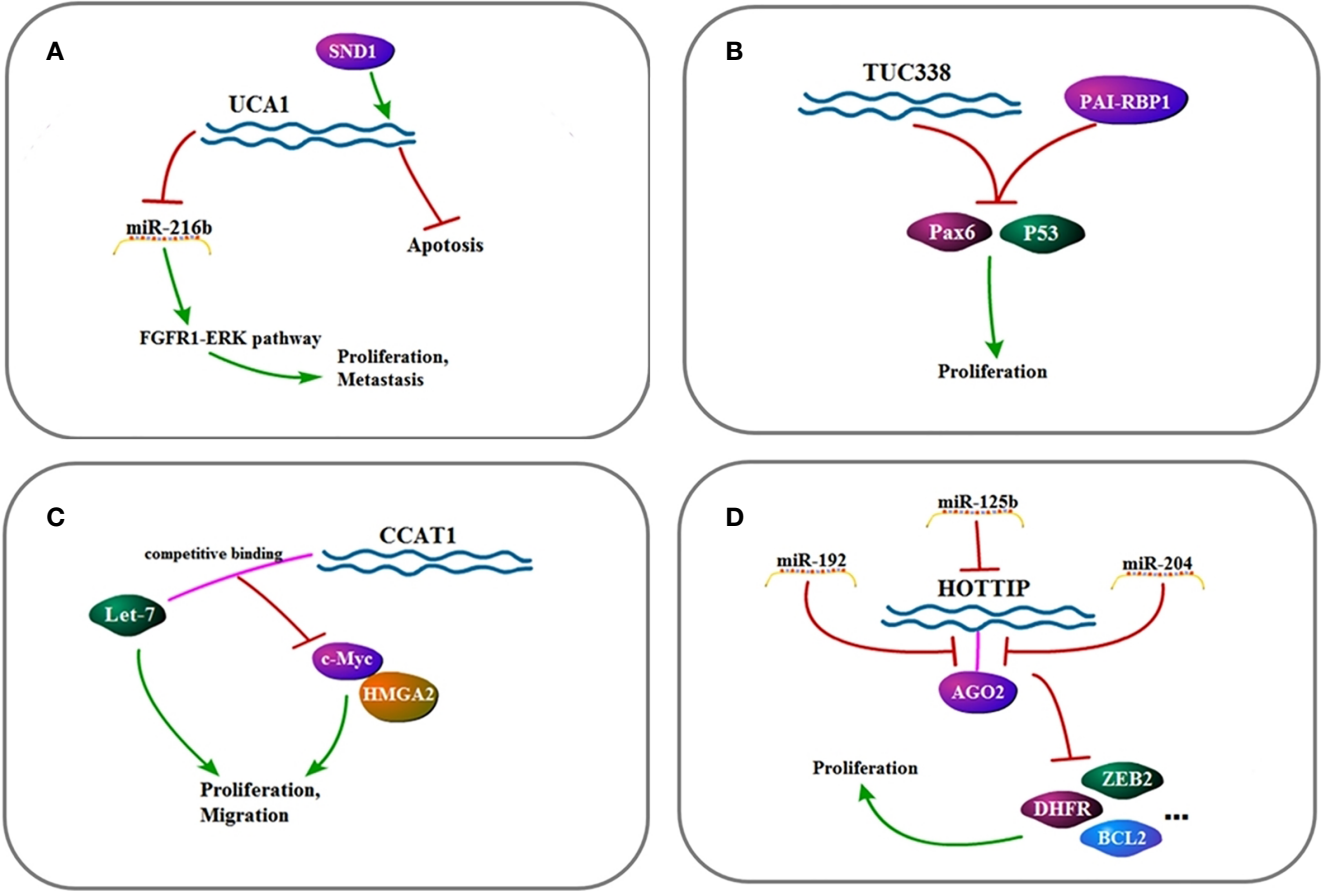
**(A)** Signaling pathway of *UCA1*: *UCA1* affects cell metastasis, proliferation, and apoptosis by up-regulating FGFR1–ERK pathway. **(B)** Signaling pathway of *TUC338*: *TUC338* affects cell proliferation by down-regulating Pax6 and P53. **(C)** Signaling pathway of *CCAT1*: *CCAT1* affects cell proliferation and migration by regulating *let-7*, c-Myc, and HMGA2. **(D)** Signaling pathway of *HOTTIP*: *HOTTIP* affects cell proliferation by down-regulating AGO2.

**Figure 4:**
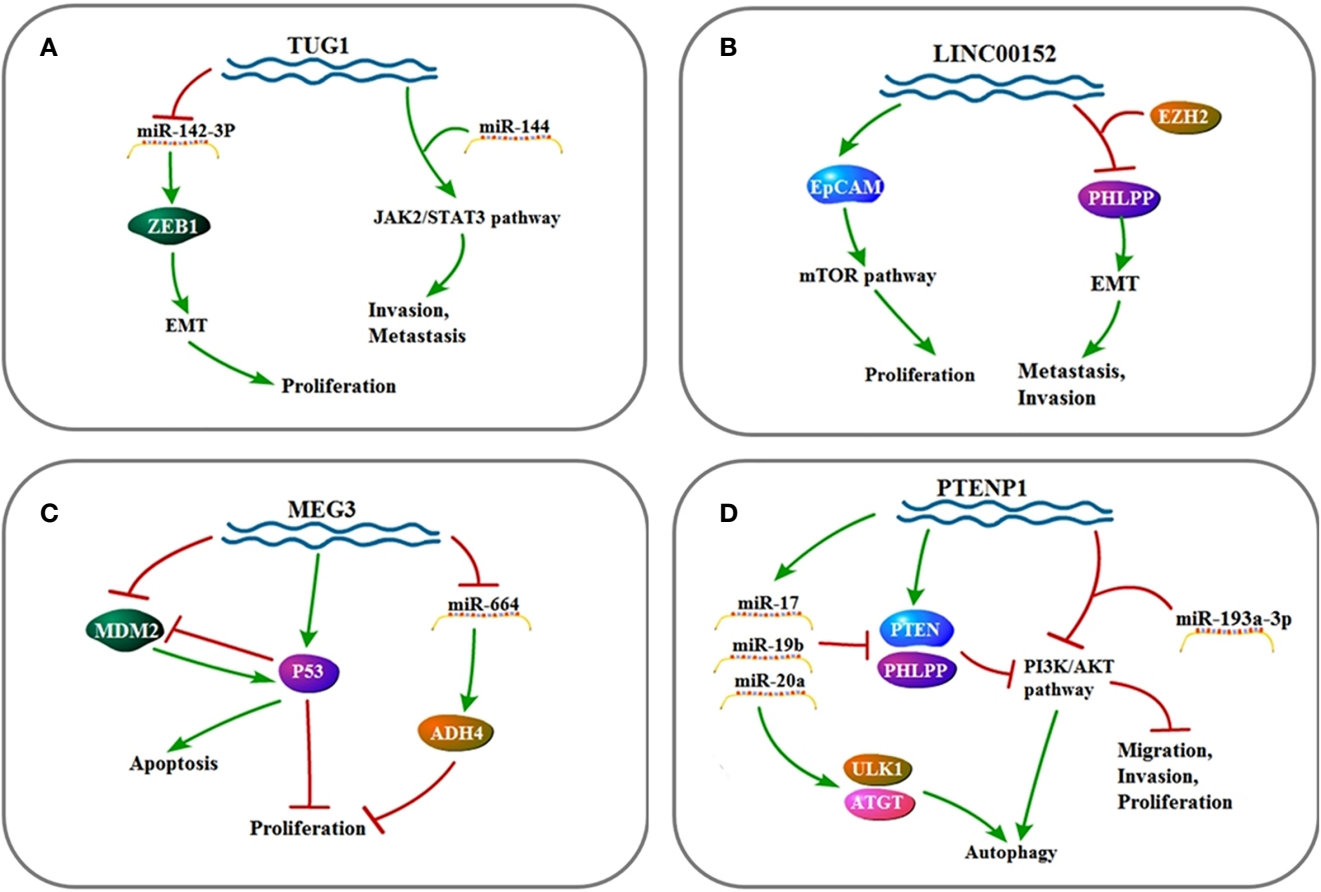
**(A)** Signaling pathway of *TUG1*: *TUG1* affects cell invasion, metastasis, and proliferation by up-regulating EMT and JAK2/STAT3 pathway. **(B)** Signaling pathway of *LINC00152*: *LINC00152* affects cell invasion, metastasis, and proliferation by up-regulating EMT and mTOR pathway. **(C)** Signaling pathway of *MEG3*: *MEG3* affects cell proliferation and apoptosis by up-regulating P53 and ADH4. **(D)** Signaling pathway of *PTENP1*: *PTENP1* affects cell invasion, proliferation, migration, and autophagy by up-regulating *miR-17, miR-19b, and miR-20a* as well as down-regulating the PI3K/AKT pathway.

In this review, we discussed a variety of lncRNAs that are proved to involve in HCC (**Figures 2**–**4**). In the past few years, a series of studies have shown the essential role of lncRNAs on cell proliferation, invasion, migration, and therapy resistance through diverse signaling pathways and molecules. Based on current studies, lncRNAs are expected to be a marker for tumor diagnosis, prognosis, and expected therapeutic effects, while lncRNA-targeted drugs still have a long way to go. Especially, considering that lncRNAs are related to the sensitivity of HCC radiotherapy and chemotherapy, lncRNAs can be used as a molecular marker to predict the clinical treatment effect of therapy treatment, and it can also be used as a target in conjunction with chemotherapy or radiotherapy to increase treatment sensitivity. Our ideal expectation for lncRNA-targeted drugs is that lncRNA-targeted drugs will serve as independent targeted drugs to treat HCC, or they will be used as adjuvant drugs to increase the efficacy of existing chemotherapeutic drugs. First, given that more than a single lncRNA is confirmed to function in HCC, to make sure which type plays a leading role in development of HCC is fatal for further research. Not only in HCC, in tumors of various tissue sources, the tissue specificity of lncRNAs is also indispensable. If future research studies prove that it is unfeasible to aim at a single lncRNA to achieve better effect, perhaps using drug that has a targeted effect on several major lncRNAs with a certain commonality based on clinical research and individual differences in patients, or employ these drugs as adjuvants can be the solutions. Also, existing studies have shown that almost each lncRNA found is involved in a variety of cell signaling pathways, but these studies are far from sufficient. Moreover, the functions of lncRNAs remained undetected, which is also a burning question. Finally, due to the structural particularity of lncRNA molecules and the characteristics of being easily degraded, how to target lncRNA molecules is also an urgent problem to be solved. With further studies, the clinical transformation of lncRNA is bound to be more mature, bringing more effective treatments to patients suffering from HCC, and contributing to the fight against cancer in humans.

## Data Availability Statement 

The original contributions presented in the study are included in the article/supplementary material. Further inquiries can be directed to the corresponding author.

## Author Contributions

XL and XX wrote the review article. RW reviewed the manuscript. All authors contributed to the article and approved the submitted version.

## Funding

This project was supported by grants from the National Natural Science Foundation of China (81472266 and 81772995) and the Excellent Youth Foundation of Jiangsu Province (BK20140032).

## Conflict of Interest

The authors declare that the research was conducted in the absence of any commercial or financial relationships that could be construed as a potential conflict of interest.
